# Adipose-derived stem cells promote survival, growth, and maturation of early-stage murine follicles

**DOI:** 10.1186/s13287-019-1199-8

**Published:** 2019-03-21

**Authors:** Lisa J. Green, Hong Zhou, Vasantha Padmanabhan, Ariella Shikanov

**Affiliations:** 1 Department of Obstetrics and Gynecology, University of Michigan, Ann Arbor, MI USA; 2 Present Address: Department of Obstetrics and Gynecology, University of South Carolina School of Medicine, Greenville, SC USA; 3 Department of Biomedical Engineering, University of Michigan, 2126 Lurie Biomedical Engineering Building, 1101 Beal Ave., Ann Arbor, MI 48109 USA; 4 Department of Pediatrics, University of Michigan, Ann Arbor, MI USA; 5 Department Molecular and Integrative Physiology, University of Michigan, Ann Arbor, MI USA; 6 Department of Environmental Health Sciences, University of Michigan, Ann Arbor, MI USA; 7 Department of Macromolecular Science and Engineering, University of Michigan, Ann Arbor, MI USA

**Keywords:** Ovarian follicle, Adipose-derived stem cells, 3D culture

## Abstract

**Background:**

Premature ovarian insufficiency is a common complication of anticancer treatments in young women and girls. The ovary is a complex, highly regulated reproductive organ, whose proper function is contingent upon the bidirectional endocrine, paracrine, and autocrine signaling. These factors facilitate the development of the follicles, the functional units of the ovary, to progress from the gonadotropin-independent, paracrine-controlled early stage to the gonadotropin-dependent, endocrine-controlled later stage. We hypothesized that the low survival rate of individually cultured early-stage follicles could be improved with co-culture of adipose-derived stem cells (ADSCs) that secrete survival- and growth-promoting factors.

**Materials and methods:**

Ovarian follicles ranging from 85 to 115 μm in diameter, from 10- to 12-day-old B6CBAF1 mice were mechanically isolated and co-encapsulated with ADSCs within alginate-based 3D culture system. The follicles were cultured for 14 days, imaged using light microscopy every 2 days, and matured at the end. Follicle media were changed every 2 days and collected for hormone measurements. Follicle diameter, morphology, number of transzonal projections, and survival and maturation rates were recorded. Statistical analyses using one- and two-way ANOVA were performed to compare hormone levels, survival of the follicles and ADSCs, oocyte maturation rates, and follicle growth.

**Results:**

The co-encapsulation of the follicles with ADSCs increased follicle survival, ranging from 42.4% for the 86–95 μm to 86.2% for the 106–115-μm follicle size group. Co-culture also improved the follicle growth, the rate of antrum formation and oocyte maturation compared to the follicles cultured alone. The levels of androstenedione, estradiol, and progesterone of co-encapsulated follicles increased progressively with time in culture.

**Conclusions:**

To our knowledge, this is the first report of an in vitro system utilizing mouse adipose-derived stem cells to support the development of the mouse follicles. Our findings suggest that co-encapsulation of ADSCs with early-stage follicles supports follicular development, through secretion of cytokines that promote follicular survival, antrum formation, and meiotic competence. The unique 3D culture system that supports the survival of both cell types has translational implications, as ADSCs could be used as an autologous source for in vitro maturation of early-stage human follicles.

**Electronic supplementary material:**

The online version of this article (10.1186/s13287-019-1199-8) contains supplementary material, which is available to authorized users.

## Background

Medical science has advanced greatly in its potential to treat various cancers, with some cancer therapies reaching > 90% 5-year survival [1]. The success of these therapies comes at the cost of future fertility and reproductive function. Cancer survival now means facing another challenge, consideration for quality of life after treatment, which may occur at the cost of future fertility and reproductive endocrine function. The preservation of oocytes, sperm, gonadal tissue, or embryos before initiation of chemotherapy is the most common fertility preservation options [2–5]. However, these options are non-applicable or contraindicated in pre-pubertal patients with hematologic malignancies who are too young to produce mature eggs or due to the concern of reintroducing malignant cells after ovarian tissue autotransplantation [4, 6, 7]. The goal of providing broad and inclusive fertility preservation options to these young cancer survivors has fueled and shaped research within the field of oncofertility [8–10]. Innovations in stem cell differentiation and in vitro gamete culture are showing great promise, and several large academic centers are cryopreserving gonadal tissue with the hope of future scientific discovery [8–13].

The human ovary is a complex, highly regulated reproductive organ, whose proper function is contingent upon the vital local and systemic bidirectional signaling [14, 15]. The functional unit of the ovary is the follicle, which relies on endocrine, paracrine, and autocrine factors to allow the successful developmental progression of the follicles from the gonadotropin-independent primary stage to the later antral stages and peaks with the maturation of fertilizable oocytes. Over the past 15 years, significant progress has been made in creating culture systems that support preovulatory follicle development and the production of meiotically competent eggs that result in live birth in rodents [16–22]. Three-dimensional (3D) hydrogel-based culture systems help support the morphologic and functional environment of the follicular unit; allow bidirectional diffusion of nutrients, hormones, and oxygen; and maintain the spherical structural integrity, thus separating and maintaining gradients of factors between cumulus and mural granulosa cells, similar to that occurring in vivo.

One of the most studied materials for 3D follicle culture is seaweed-derived alginate [23–27]. Alginate is an inert polysaccharide polymer that crosslinks at physiological conditions and supports culture, survival, and maturation of secondary follicles from different species [16, 26, 28–30]. However, culture and maturation of early-stage follicles from mice with an initial diameter below 120 μm have long presented challenges for successful folliculogenesis, survival, and maturation [31]. When cultured individually at this early stage, the follicles show markedly decreased survival. Co-culture of early-stage ovarian follicles with “feeder cells,” such as embryonic fibroblasts, multiple ovarian follicles, or ovarian stromal cells, have been found to significantly improve the outcomes of follicular development within these in vitro systems, possibly through the interactions of multiple paracrine signals. However, limited sources of human embryonic fibroblasts or ovarian stromal cells, and incompatibility of human follicles with mouse cells limit the translation of this approach to patients [32, 33]. Therefore, the accessibility, compatibility, and multipotent differentiation potential of adipose-derived stem cells (ADSCs) make them attractive candidates for human translational studies and future therapeutic indications [34]. The use of ADSCs and other types of mesenchymal stem cells has been well studied for their paracrine-mediated therapeutic effects on wound healing and regeneration [35–38]. Recent reports of the use of mesenchymal stem cells for the paracrine mediated, restoration of ovarian function in animal models of primary ovarian insufficiency [39–41] provide the basis for our hypothesis that the paracrine signals from ADSCs can rescue individually cultured primary and early secondary ovarian follicles and promote folliculogenesis [38]. We recently analyzed the secretome of human ADSCs cultured in three-dimensional culture and found statistically significant increases in cytokine production [36] and gene expression [38] among genes and cytokines that have previously been established as vital to follicular survival, growth, and development. Upregulated genes included vascular endothelial growth factor A (VEGFA), transforming growth factor-beta 2 (TGF-β2), and hepatocyte growth factor (HGF), which are known to be beneficial for follicular angiogenesis, follicular regulation, and apoptotic suppression, respectively. Zhou et al. demonstrated an increase in monocyte chemotactic protein-1 (MCP-1)  (related to oocyte maturation), Follistatin (regulation of granulosa cell proliferation), and IL-6 (regulator of cumulus cell function) [36].

In this study, mouse ovarian follicles were co-encapsulated with ADSCs in alginate hydrogels and cultured for 14 days. Follicle survival, growth, and maturation rates significantly improved in the presence of ADSCs. To our knowledge, this is the first report of the application of mouse ADSCs supporting in vitro ovarian follicular development in a 3D co-culture setting. Research in this direction could also be the basis for future research into the secretome of ADSCs within in vitro culture system to help us better understand ADSC-derived secretory factors that play vital roles in establishing optimal ovarian micro-environment for promoting early stages of folliculogenesis.

## Methods

Because ADSCs could provide a readily accessible, high yield, autologous source for in vitro follicle culture of early-stage follicles, we investigated whether ADSCs from the same species co-encapsulated with primary and early secondary ovarian follicles in a 3D alginate hydrogel system would improve the outcomes of in vitro folliculogenesis.

### Source of adipose-derived stem cells

OriCell C57BL/6 Mouse ADSCs (Cyagen, Santa Clara, CA, USA) were commercially acquired. The ADSCs were isolated from the inguinal fat pads of pre-pubertal C57B6 female mice and cultured in monolayer for six passages prior to the first use. The cells have previously tested positive for stem cell markers CD34, CD44, stem cells antigen-1 (SCA-1), and negative for CD117 by flow cytometry analysis. Upon procurement, the cells were thawed and subsequently passaged for up to three passages using Cyagen ADSC growth media (includes basal medium and growth supplement; Cyagen Biosciences Inc., Suzhou, Jiangsu, China) per manufacturer's instructions.

### Lineage differentiation and characterization of ADSCs

To determine the multipotency of the ADSCs used in the follicle co-culture, the cells were cultured in osteogenic, adipogenic, and chondrogenic media per the manufacturer’s protocol (Cyagen). All cells were imaged using light microscopy (Leica, DMI 3000, Germany).

### ADSC surface marker expression

ADSCs were immuno-stained for surface marker analysis with fluorescence-conjugated anti-mouse antibodies in accordance with the manufacturer’s instructions. Briefly, cells at 7–8th original passages were grown in monolayer until reaching 80–90% confluence. Cells were then trypsinized with 0.25% trypsin-EDTA and washed with FACS buffer (PBS and 2% BSA). Cell number was determined using an automated cell counter Moxi z-mini (Orflo Technologies, Ketchum, ID, USA). Cells (2.0 × 10^5^) were incubated with monoclonal antibodies for 30 min on ice in the dark. Unstained cells were used as a negative control. The following surface markers were analyzed: CD45/Alexa Flour, CD34/BV421, CD90/PE, CD31/APC, SCA-1/BB515, and CD44/BV570. Ten thousand events per sample were obtained for each marker, and sample analysis performed in duplicate. Data were analyzed using FlowJo (LLC, Ashland, OR, USA).

### Viability of ADSCs within the alginate hydrogel culture system

To determine the survival of ADSCs within the culture system and the effects of both culture media and exogenous hormones on cell viability, 5 × 10^5^ ADSCs/ml were suspended in alginate solution and encapsulated in crosslinked alginate beads with and without the ovarian follicles. Beads were removed at days 1, 4, 7, 10, and 14 of culture to test the viability, and the experiments were repeated four times. Using the LIVE/DEAD assay (Thermofisher, Eugene, OR, USA), alginate beads were incubated with Calcein AM and ethidium homodimer-1 per manufacturer’s protocol. Beads were then imaged with light microscopy (DMI3000, Leica, Germany). Quantification of the percentage of live and dead cells was performed via an automated analysis using ImageJ software (Rasband, W.S., ImageJ, National Institutes of Health, Bethesda, MD, USA). Light microscopy, green, and red filter images were obtained for each condition at each time point. All images were converted to a binary image in which cell pixels were black and the background was white. The images were then analyzed for the number of total cells, based on the light microscopy image, and the number of green (live) and red (dead) fluorescing cells.

### Follicle isolation, encapsulation, and culture

The ovaries were obtained from 10- to 12-day-old F1 hybrid mice (C57BL/6JRccHsd inbred ×CBA/J CrHsd, Envigo, Indianapolis, IN, USA). The animals were bred and housed in a light (12-h light/12-h darkness) and temperature-regulated environment within ventilated cages. All animal handling procedures were in accordance with the regulations and guidelines by the National Institutes of Health Guide for the Care and Use of Laboratory Animals and approved by the Institutional Animal Use and Care protocol of the University of Michigan. Primary and early secondary ovarian follicles were mechanically isolated from the ovaries and encapsulated as previously described [41] with slight modifications. Briefly, for each experiment, primary and early secondary follicles, ranging from 85 to 115 μm in diameter, were mechanically isolated from 6 to 8 ovaries using insulin gauge needles, while immersed in dissection media (Leibovitz’s L-15 medium/1% fetal bovine serum/0.5% penicillin-streptomycin). Isolated follicles from multiple mice were combined and stored in maintenance media (minimum essential media-alpha modifications (α-MEM) with GlutaMAX, 1% fetal bovine serum, and 0.5% penicillin-streptomycin) in a CO_2_ incubator for up to 2 h. Morphologically intact follicles ranging from 85 to 115 μm in size were selected and immersed in 0.25% alginate, with and without ADSCs. Droplets (10 μl) of alginate with the enclosed follicle (+/− ADSCs) were immersed in 50 mM CaCl_2_ and 140 mM NaCl solution for 2 min to crosslink and form beads. Alginate beads with encapsulated follicles with and without ADSCs were then transferred to individual wells of a 96-well plate, containing 100 μl of growth media (α-MEM supplemented with 3 mg/mL bovine serum albumin (BSA, MPBiomedicals, Irvine, CA, USA), 1 mg/mL bovine fetuin, 5 μg/mL insulin, 5 μg/mL transferrin, 5 ng/mL selenium (ITS, Sigma, St. Louis, MO, USA), and 10 mIU/mL highly purified, human-derived, follicle-stimulating hormone (FSH) (Urofollitropin, Ferring Pharmaceuticals, Saint-Prex, Switzerland). The follicles were cultured in either 0.25% alginate beads alone or co-encapsulated in 0.25% alginate with ADSCs at a concentration of 5 × 10^5^ cells/ml at 37^o^C in 5% CO_2_ for 14 days. The ADSC concentration that maximized follicle survival was determined empirically by co-encapsulating follicles with ADSCs at concentrations ranging from 1 × 10^5^ to 8.0 × 10^5^ cell/ml. Beginning on day 6 of culture, half of the medium (50 μl) from each well was replaced with fresh growth medium every 2 days and the medium was stored at − 20 °C for a subsequent measure of steroid hormone production.

### Assessment of follicle survival and growth

Follicle viability was determined based on the absence of morphological characteristics such as early oocyte extrusion, signs of atresia, or degeneration. To assess the growth of the follicles, light microscopy images were captured using an inverted Leica DM light microscope (DMI 3000, Leica, Germany). An average diameter was obtained by taking two perpendicular measurements of surviving follicles using ImageJ software (National Institutes of Health).

### Steroid hormone production by encapsulated follicles

The media from 3 to 4 follicles of the same initial follicle diameter range and time points were pooled together to achieve the requisite volume. Steroid hormone production was measured in a total of 6 pooled samples per experimental condition for each follicular size category. Concentrations of androstenedione (A4), 17β-estradiol (E2), and progesterone (P4) in the collected media were measured at the University of Virginia Center for Research in Reproduction Ligand Assay and Analysis Core (Specialized Cooperative Centers Program in Reproduction Research). The sensitivity for A4 was 0.1 ng/mL, for estradiol 10 pg/ml, and for P4 0.1 ng/ml. The coefficient of variation for A4 was 1.5% for ALPCO QC1 at the range 0.15–0.25 ng/ml, 2.6% for ALPCO QC2 at the range 1.75–2.91 ng/ml, 1.0% for Bio-Rad 1 at the range 0.42–0.72 ng/mL, 3.7% for Bio-Rad 2 at the range 0.96–1.78 ng/mL, and 1.1% for Bio-Rad 3 at the range 1.7–3.3 ng/mL. The coefficient of variation for E2 was 3.9% for Bio-Rad 1 at the range 58.0–108.0 pg/ml, 10.2% for Bio-Rad 2 at the range 176.0–328.0 pg/mL, and 1.5% for Bio-Rad 3 at the range 437.0–811.0 pg/mL. The coefficient of variation for P4 was 13.7% for Rodent QC1 at the range 8–15 ng/ml, 2.5% for Rodent QC2 at the range 2.6–4.8 ng/ml, and 3.9% for Rodent QC3 at the range 16.4–30.4 ng/mL.

### Meiotic competence of oocytes from encapsulated follicles grown in culture

Following 14 days of culture, all surviving antral follicles that reached a diameter of 250 μm or greater were removed from the alginate beads by incubating for 10 min with 50 μl of pre-warmed alginate lyase media (α-MEM with10 IU/mL alginate lyase) [MP Biomedicals, Irvine, CA, USA]. Individual follicles were aspirated from the follicle culture plate and placed in 20 μl droplets of maturation media (α-MEM supplemented with 10% fetal bovine serum, 5 ng/mL epidermal growth factor, 1.5 IU/mL human chorionic gonadotropin, and 10 mIU/mL FSH) for 16–18 h. After incubation, the expanded cumulus granulosa cells were removed from the oocytes by adding 0.5 μl of 0.3% hyaluronidase to each droplet. Oocytes were aspirated, and the remaining adherent granulosa cells were removed mechanically by pipetting. Images were taken at × 400 using light microscopy, and the follicles were classified as a germinal vesicle (GV), MI, and MII or degenerated based on their morphology.

### Quantification of transzonal projections

To assess the extent of transzonal projections—membraneous extensions from the granulosa cells of the ovarian follicles to the oocyte through the zona pellucida that facilitates exchange between the two cells—the follicles encapsulated in 0.25% alginate with and without ADSCs were cultured for 2 days. Following 2 days of culture, the follicles were removed from the alginate after a 20-min incubation in 50 μl of alginate lyase media (α-MEM with 10 IU/mL alginate lyase). The follicles were then aspirated and fixed with 4% paraformaldehyde at room temperature for 1 h. The follicles were stained overnight for F-actin (1:50 Molecular Probes, Invitrogen) and DNA (Hoechst 33342; Invitrogen) at 4 °C on a plate shaker. Using a Nikon A-1 microscope, a 10-μm Z stack image was obtained for ten follicles, from each follicle size category, encapsulated with and without ADSCs. Using ImageJ, an intensity profile was created by drawing four pixel lines in the zona pellucida surrounding the oocyte. Background intensity was determined for each individual follicle, and the frequency of TZPs was determined by the number of peaks in the profile above the background threshold.

### Statistical analysis

Statistical analyses were performed using GraphPad Prism 7 (GraphPad Prism Software, La Jolla, CA, USA). For all statistical analyses, a *p* value less than 0.05 was considered significant. Specifically, survival data was analyzed using the built-in Kaplan-Meier survival curve analysis. One-way analysis of variance (ANOVA) was performed for ADSC survival and maturation data. Two-way ANOVA were performed for follicle growth and TZP counting data to account for the effect of culture time and/or initial follicle sizes. Sidak’s multiple comparisons test was sequentially performed where appropriate. Hormone data was analyzed using one-way ANOVA with repeated measures, followed by Tukey’s multiple comparison tests to determine significant changes of hormone levels over time within each follicle group.

## Results

### ADSCs maintain their viability and stemness within the hydrogel

**Fig. 1:**
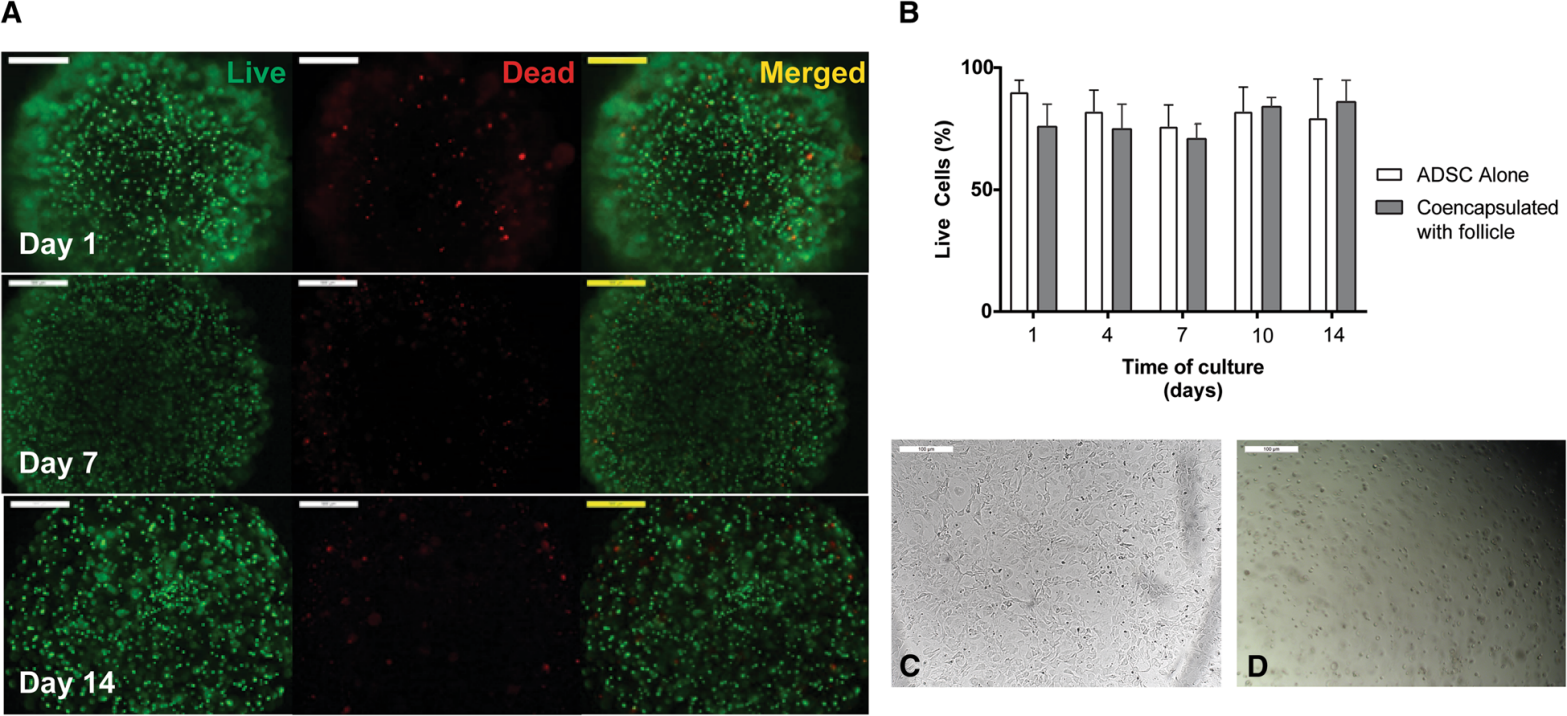
Encapsulation of ADSC in alginate. **a** Representative live/dead images of ADSCs in alginate over 14 days of culture in follicle culture conditions. Scale bars = 500 μm. **b** No significant difference was found in ADSC survival in when compared to traditional two-dimensional culture over the 14-day culture period. Sample size, *n* = 3. **c** Representative image of ADSC cultured in traditional tissue culture-treated flask. Scale bars = 100 μm. **d** Representative image of ADSC cultured in alginate. Scale bars = 100 μm

Assessment of viability using LIVE/DEAD assay (Molecular Probes) revealed that the majority (between 75 and 95%) of ADSCs maintained their viability in the alginate hydrogel system (Fig. 1a, b) and was not affected by the presence of the follicles. Live cells reacted with the Calcein AM and fluoresced green, while the dead cells stained red. Only a small percentage of cells were dead following 14 days of culture, which demonstrated that the alginate system implemented for the follicle culture supported stem cell viability. When cultured at the bottom of the flask, the ADSCs demonstrated a fibroblast-like morphology, adhered to the bottom of the plastic flasks, and expanded in monolayer culture (Fig. 1c), thus meeting the standards set forth for human-derived adipose stem cells [42]. ADSCs encapsulated in alginate remained circular for the duration of the culture (Fig. 1d). Successful induction of ADSCs into adipogenic, osteogenic, and chondrogenic lineages further confirmed the stemness of the cells (Additional file 1: Figure S1A).

### ADSCs continue to express mesenchymal cell-specific surface markers after 4 days of culture encapsulated in alginate

Flow cytometry analysis of the cells after 1 and 4 days of cell culture confirmed that the cells expressed SCA1, CD34 (a surrogate marker for hematopoietic stem and progenitor cells), and CD44 (a cell surface glycoprotein involved in cell-cell interactions, cell adhesion, and migration), but were negative for CD45 (hematopoietic marker) and CD31 (endothelial marker) (Additional file 1: Figure S1B; day 0 and day 4 of culture). The fact that these cells continue to express mesenchymal stem cell-specific antigens after 4 days of culture in the alginate bead indicates that they met (1) the minimum criteria set forth by the International Society for Cell Therapy for mesenchymal stem cells upon purchase and (2) continue to demonstrate the phenotypic expression of stem cells during the gonadotropin-independent stage of early folliculogenesis.

### Co-encapsulation of primary and early secondary follicles with ADSCs improves in vitro follicular growth and development

**Fig. 2:**
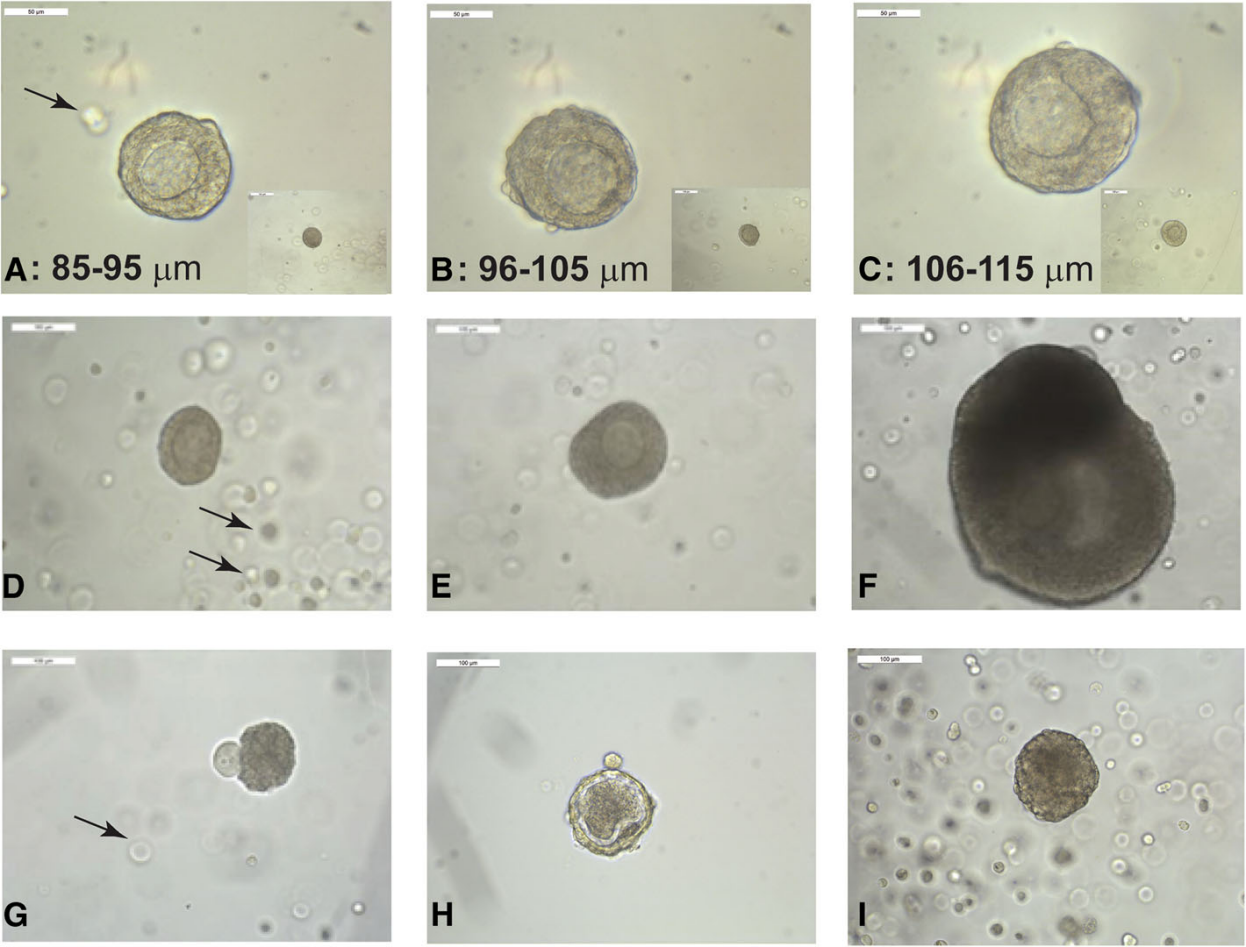
Representative images of the follicles in culture. Arrows = individual ADSCs in alginate. **a**–**c** Representative images of the follicles in vitro from the three diameter groups (*A* = 85–95 μm, *B* = 96–105 μm, *C* = 106–115 μm). Scale bars = 50 μm (100 μm for inserts). **d**–**f** Representative images of follicle growth and follicle development on days 2 (**d**), 4 (**e**), and 12 (**f**). Scale bars = 100 μm. **g**–**i** Representative images of various modes of follicular death in vitro. **g** Extruded oocyte. **h** An atretic-looking follicle. **i** A dark and dense looking follicle. Scale bars = 100 μm

After isolation, based on the average initial diameters of the follicles on day 0, the follicles were grouped into three classes, small (85–95 μm), medium (96–105 μm), and large (106–115 μm) (Fig. 2a–c). The follicles in the 85–95-μm range represented primary follicles characterized by an oocyte surrounded by a single layer of granulosa cells. The follicles in the 96–105-μm range had 1–2 layers of granulosa cells. The follicles in the 106–115-μm range had > 2 layers of granulosa cells. The average starting follicle diameter for the three groups ranged between 90 + 3.15 μm, 100 + 2.75 μm, and 110 + 2.64 μm. The typical growth pattern and antrum formation of the primary ovarian follicle co-encapsulated with ADSCs in an alginate bead is demonstrated in Figs. 2d, e. The follicles that did not grow and reach antral stages usually extruded their oocyte (Fig. 2g) or had an abnormal phenotype without the clear separation of the oocyte from the granulosa cells (Fig. 2h). In some cases, the follicles continued to grow without the presence of a viable oocyte (Fig. 2i).

Initial studies focused on optimizing the number of ADSCs for encapsulation with the follicles. Co-encapsulation with 1.0 × 10^5^, 3.0 × 10^5^, 5.0 × 10^5^, and 8.0 × 10^5^ ADSC/mL showed marked improvement in follicle survival, although survival rate differed between the four concentrations (Additional file 2: Figure S2 A, C, E). Co-encapsulation of the follicles in all sizes with ADSCs at a concentration of 1.0 × 10^5^ did not contribute to improved survival, while 3.0 × 10^5^, 5.0 × 10^5^, and 8.0 × 10^5^ were better than 1.0 × 10^5^ at least in one or more ranges. Although optimal survival of the follicles in the range of 95–105 μm and 105–114 μm was achieved when they were co-encapsulated with 8.0 × 10^5^ cells/mL, primary follicles with diameters ranging between 85 and 95 μm demonstrated best survival (50%) when co-cultured with 5.0 × 10^5^ cells/mL for 14 days (Additional file 2: Figure S2A). Since the concentration of 5.0 × 10^5^ cells/mL maximized the survival of the follicles in the 85 and 95-μm primary follicle range and demonstrated improved survival across the board, it was identified as the concentration for co-encapsulation of ADSCs for further experiments and used for all subsequent studies.

**Fig. 3:**
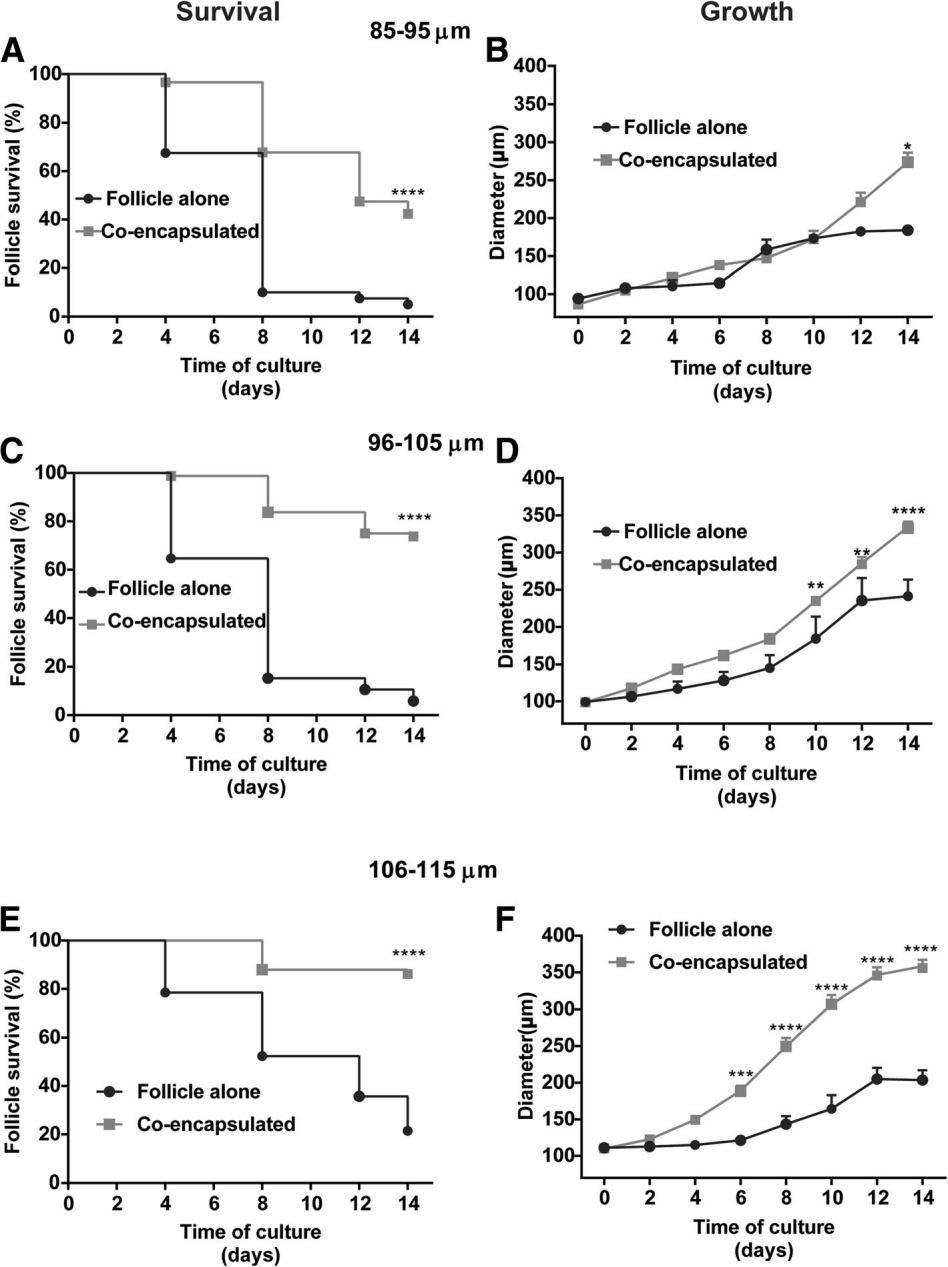
Survival and growth of the follicles with and without co-encapsulation with ADSCs. *p < 0.05, **p < 0.01, ***p < 0.001, ****p < 0.0001. Survival and growth of the follicles in the ranges of 85–95 μm (**a**, **b**), 96–105 μm (**c**, **d**), and 106–115 μm (**e**, **f**) cultured alone (gray) or co-encapsulated with ADSCs (black). Sample sizes of growth curves: 85–95 μm: *n* = 2 (follicle alone) and 25 (co-encapsulated); 96–105 μm: *n* = 5 (follicle alone) and 59 (co-encapsulated); and 106–115 μm: *n* = 9 (follicle alone) and 50 (co-encapsulated). Data in growth curves presented as mean ± SEM

Survival rates of the follicles co-encapsulated with 5.0 × 10^5^ ADSC/mL were significantly greater for all three follicle size classes when compared with the follicles cultured alone (*p* < 0.0001). The survival rate of the follicles co-encapsulated with 5.0 × 10^5^ ADSCs/mL following the 14-day culture was 42.4% for the follicles in the initial diameter range of 85–95 μm, 73.8% for the 96–105-μm-diameter range, and 86.2% for the 106–115-μm-diameter range (Fig. 3, left panels). Only 5, 5.9, and 21.4% of the follicles of size classes 85–95, 96–105, and 106–115 μm survived when cultured in the absence of ADSCs. Significantly fewer follicles in the small (85–95 μm) diameter range survived, even after co-encapsulation with ADSCs, compared to the medium (96–105 μm, *p* = 0.0002) and the large (106–115 μm, *p* < 0.0001) diameter groups. The survival rate of the medium and the large diameter follicle groups did not differ from each other (*p* = 0.0812).

All tested concentrations of ADSCs supported follicular growth to a greater extent compared to those cultured in the absence of ADSCs (Additional file 2: Figure S2 B, D, F). Following the 14-day culture of the follicles co-encapsulated with 5.0 × 10^5^ cells/mL, which was the optimal cell number chosen, there was a statistically significant improvement in follicular growth on culture day 14 for co-encapsulated follicles of an initial diameter of 85–95 μm (*p* = 0.0165) (Fig. 3, right panels). In co-encapsulated follicles of initial diameter 96–105 μm, there was a statistically significant increase in the follicle size starting on culture day 10 (*p* = 0.0055), which continued through culture days 12 (*p* = 0.0063) and day 14 (*p* < 0.0001). Co-encapsulated follicles of the largest initial follicle size, 106–115 μm, reached the larger size, as early as culture day 6 (*p* = 0.0007), which continued for the duration of culture (*p* < 0.0001 for days 8 to 14). The final follicular sizes of the follicles co-cultured with 5.0 × 10^5^ ADSCs/mL were 273.6 ± 12.7 for the 85–95 μm, 334.3 ± 7.3 for the 96–105 μm, and 358.2 ± 8.9 for the 106–115-μm follicle classes, which were all significantly greater than those cultured in the absence of ADSC.

### Steroidogeneic potential of the follicles encapsulated with ADSCs

**Fig. 4:**
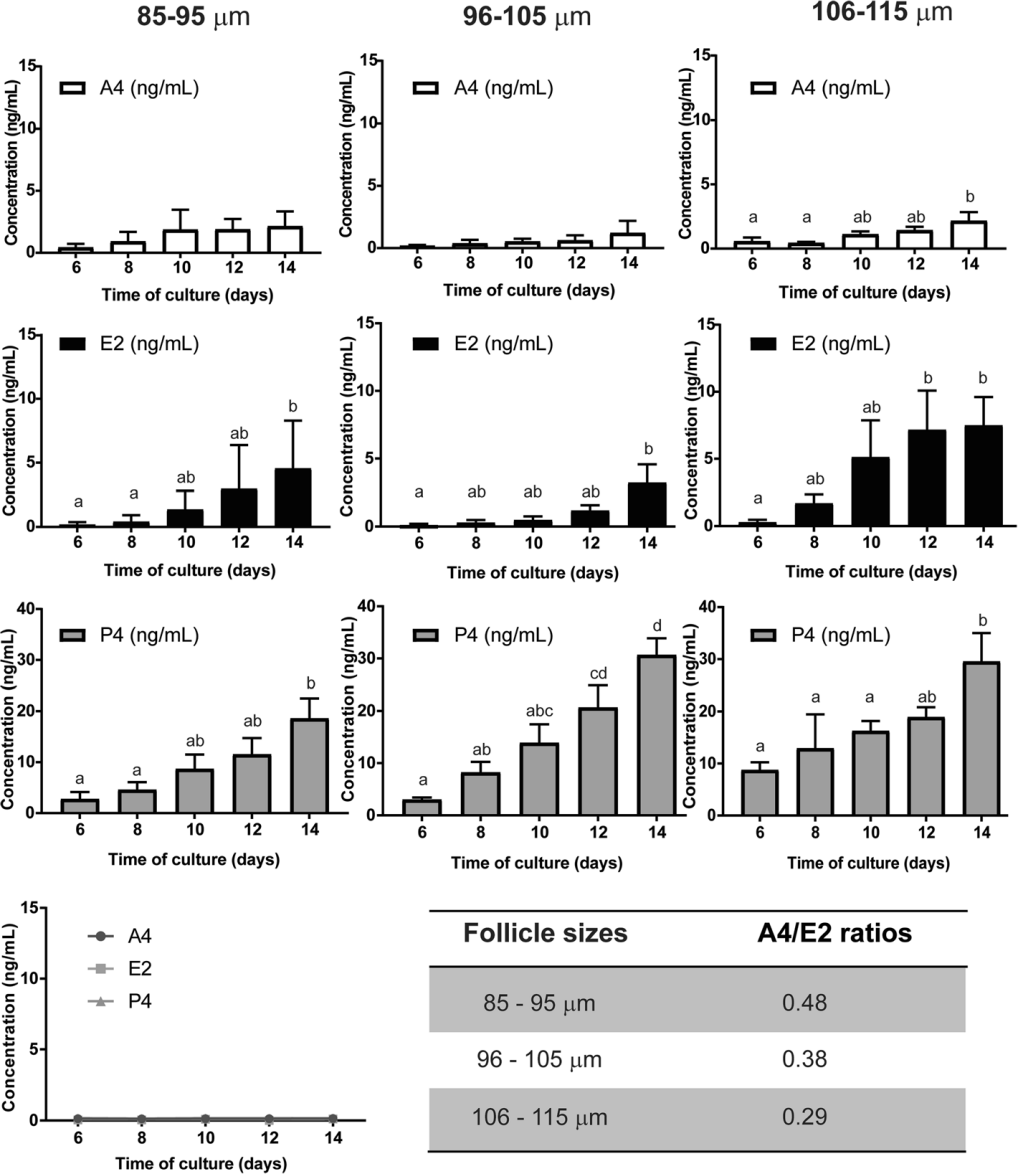
Hormone secretion profiles of the follicles co-encapsulated with ADSCs and ADSCs only. Increasing hormone production during culture suggests follicular function. Top row demonstrates the levels of A4, second row E2, and third row P4. Statistical significance was marked by different letters. The hormone secretion profiles of ADSCs when encapsulated alone in alginate are demonstrated in the figure on the bottom left. Sample size: *n* = 3 per follicle size group. Data presented as mean ± SEM. A4/E2 ratios on the last day of culture are summarized in the table

As the functional unit of the ovary, the follicles are essential not only for their reproductive capabilities, but also for their endocrine function as well. The steroid production by the follicles co-cultured with ADSCs was used as an index to confirm that the follicles perform their steroidogenic role. The follicles cultured alone without ADSCs did not survive in sufficient numbers to analyze their steroidogenic activity for the whole duration of the culture. Levels of A4 in all the conditions, 85–95 μm, 96–105 μm, and 106–115 μm co-encapsulated with ADSCs, showed a progressive increase with time in culture with levels on D14 significantly greater in the 106–115 group compared to D6 (*p* = 0.0300) and D8 (*p* = 0.0201) (Fig. 4, top row). Across all follicular classes, A4 at all time points were significantly greater than ADSCs cultured alone. Levels of E2 also showed a progressive increase starting on day 8 and reaching maximum concentration on day 14 for all three follicular diameter classes. In the 85–95-μm follicular group, D14 E2 levels were significantly higher than that produced on D6 (*p* = 0.0287) and D8 (*p* = 0.0401). In the 96–105-μm group, D14 E2 levels were significantly higher compared to D6 (*p* = 0.0450). The levels of E2 in the 106–115-μm group were significantly higher on D12 (*p* = 0.0435) and D14 (*p* = 0.0346) of culture compared to the D6 group (Fig. 4, second row).

Levels of P4 levels, similar to A4 and E2, also showed an increase starting on day 8 (Fig. 4, bottom panel). Levels of P4 on D14 of culture averaged 18.63, 30.68, and 29.56 ng/ml respectively for the 85–95, 96–105, and 106–115-μm follicular groups. While P4 levels on D14 in the 85–95 μm follicular group was only significantly higher than that of D6, the D14 levels in the 96–105 and 106–115-μm follicular groups were significantly higher than seen on D6, D8, and D10 (Fig. 4, 3rd row).

The production of sex hormones A4, E2 and P4 by the ADSCs were close to detection limit compared to the levels secreted by the follicles and remained so for the duration of the culture (Fig. 4, bottom row).

The androgen/estrogen ratio used to assess aromatase (CYP19a1) activity [43–45] was 0.48, 0.38, and 0.29, respectively, for the small, medium, and large follicle groups (Fig. 4). A4/E2 ratio below or close to 1 suggests appropriate quantities of steroid biosynthesis. Increase in androgen/estrogen ratio would indicate an androgenic intra-follicular milieu and less favorable environment for follicle development and oocyte maturation, while low ratios indicate appropriate interactions between theca and granulosa cells that results in the conversion of A4 to E2.

### Oocytes from the follicles co-cultured with ADSCs are meiotically competent

**Fig. 5:**
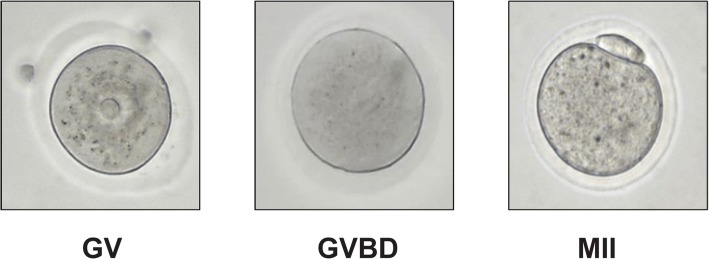
Representative images of oocyte stages after in vitro follicle maturation. GV germinal vesicle in an immature oocyte, GVBD germinal vesicle breakdown, MII oocytes arrested at meiosis II stage

In vivo, in response to LH, oocytes resume the meiotic process and undergo germinal vesicle breakdown, and progress through metaphase 1 (MI) and metaphase 2 (MII) stages (Table 1). Morphologically, the progression from MI to MII is determined when meiotically competent oocytes extrude the polar body and resume meiosis [28, 46]. The follicles co-encapsulated with ADSCs were able to resume meiosis and undergo in vitro maturation. A representative example is shown in Fig. 5. In vitro maturation rate of the antral follicles in the 85–95-μm group was 87.5%, in the 96–105-μm group was 93%, and in the 106–115-μm group was 95%. None of the follicles from all three classes when cultured without the ADSCs were able to resume meiosis. This finding suggests that the follicles co-cultured with ADSCs were able to produce meiotically competent oocytes.

**Table 1:** Quantification of follicular in vitro maturation

Initial follicle size (μm)	Follicles, *n*	MII, *n* (%)	MI, *n* (%)	GV, *n* (%)	DG, *n* (%)
85–95	24	11 (46)	10 (42)	1 (4)	2 (8)
96–105	55	29 (53)	21 (38)	1 (2)	4 (7)
106–115	36	18 (50)	16 (44)	0 (0)	2 (6)

### Co-encapsulation with ADSCs is associated with an increased number of transzonal projections

**Fig. 6:**
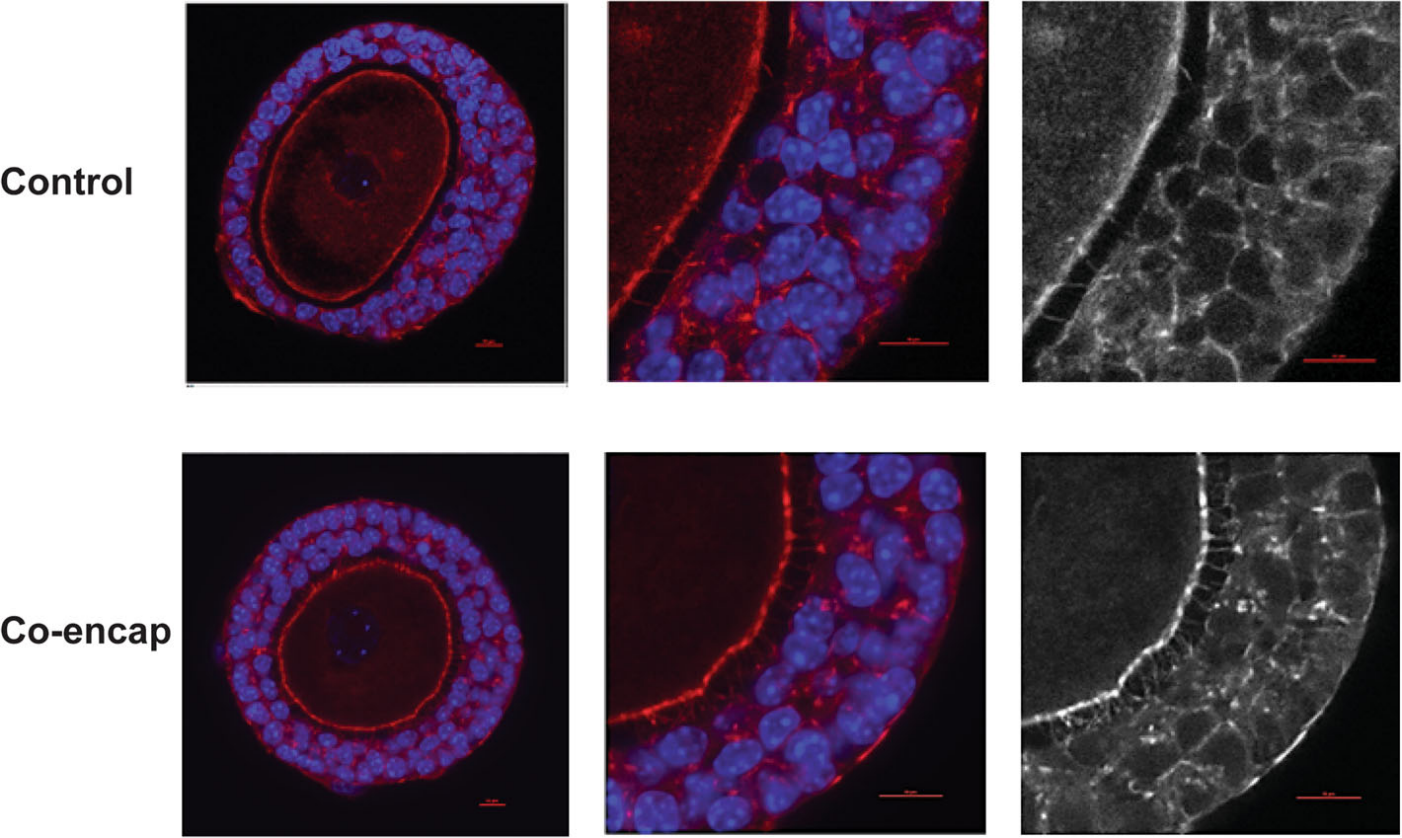
TZP counts in the follicles cultured alone and co-encapsulated with ADSCs. Significantly higher numbers of TZPs are found between the oocyte and cumulus granulosa cells of the follicles co-encapsulated compared to control after 2 days of culture. Scale bars = 10 μm. Sample size: *n* = 10 per follicle size group per condition

Transzonal projections (TZPs) are actin-rich, filamentous, cellular extensions that project from the granulosa cell, transverse the zona pellucida, and terminate on the oocyte cell surface (Fig. 6) [46, 47]. Hornick et al. have found that TZP measurements taken on day 2 of the follicle culture are predictive of follicle integrity [31]. They reported that the follicles cultured individually had less transzonal projections than the follicles co-cultured in groups and decreased numbers of TZPs were associated with decreased follicle survival and oocyte quality. Consistent with this report, in our studies, the follicles from all three size classes co-encapsulated with ADSCs had a significantly higher number of TZPs between the oocyte and cumulus granulosa cells relative to those not co-cultured with ADSCs (Table 2).

**Table 2:** Quantification of TZP counts (per 10-μm slice) in the follicles cultured alone and co-encapsulated with ADSCs

Initial follicle size (μm)	Follicle alone	Co-encapsulated with ADSCs
85–95	3.06 ± 0.82	5.76 ± 1.70****
96–105	3.38 ± 0.8	7.47 ± 1.18****
106–115	4.13 ± 0.8	8.20 ± 1.46****

Sample size, *n* = 10 per follicle group. ****p < 0.0001

## Discussion

Findings from these studies indicate that co-encapsulation of the follicles with ADSCs promotes follicular survival, growth, and steroidogenesis. The beneficial effects of ADSCs in promoting early follicular growth and function are discussed below.

Prior studies have examined and established that early-stage ovarian follicles undergo significant atresia, and although activated, do not grow when cultured individually [31–33]. Co-culture of early-stage follicles with “feeder cells,” such as embryonic fibroblasts, stromal-thecal cells, or mesenchymal stem cells, have been proposed to improve the outcomes of in vitro folliculogenesis. Yet, each of these previously established “feeder cell” introduces certain limitations making them less attractive for follicle culture especially in the context of human translation. For example, co-culture with embryonic fibroblast would necessitate the use of cells from an allogeneic or xenogeneic source. The use of stromal-thecal cells as the source from the frozen ovarian tissue presents challenges, because of the quality and purity of the cells. Some promise was demonstrated with human mesenchymal cells derived from the bone marrow that have been co-cultured with early-stage human follicles [42]. However, because the follicles were cultured in groups and not grown to the antral stage, the potential benefit of this approach relative to the approach used in our study cannot be ascertained. To improve the outcomes of the primary follicle culture, the use of ADSCs present a preferable tissue because they can be obtained from the same donor as the ovarian tissue, tissue acquisition is less invasive compared to MSCs, and the cells can be harvested in larger amounts when compared to other stem cell sources, such as the bone marrow [48].

While the entirety of the beneficial secretome remains unknown, ADSCs have been shown to secrete factors important for follicular development such as HGF (hepatocyte growth factor), VEGF (vascular endothelial growth factor), PGF (placental growth factor), TGF-β (transforming growth factor-beta), LIF (leukocyte inhibiting factor), and IGF (insulin-like growth factor) [35, 49, 50]. The addition of these factors alone has not supported folliculogenesis in vitro, possibly because of the dynamic bidirectional interactions between the follicles and the ADSCs that cannot be replicated by adding specific components exogenously to the culture well. The common link in the secretome and temporal signature of these different paracrine secreting cells may hold the answer to this question requiring a co-culture system for provision of a full repertoire of paracrine factors. These findings from this study that co-encapsulation with ADSCs provided better support for follicle survival, improved follicle growth and function relative to the follicles cultured alone, are consistent with this premise. Our finding that optimal outcome was not achieved with low and high (> 500,000cells/mL) concentrations of ADSCs point to the need not only for appropriate paracrine factors but also for appropriate levels of paracrine factors.

Co-encapsulation and co-culture of ADSCs contributed to successful oocyte maturation at the end of 14-day long folliculogenesis that is evidenced by the fact that oocytes cultured within our system were able to resume meiosis, with > 90% of antrum forming follicles undergoing germinal vesicle breakdown, and 40–55% extruding the first polar body and entering into the second stage of meiosis. Our results are similar to previously reported studies in early-stage follicles [31–33] but lower than the rates seen for secondary follicles [16, 51]. Similar to previous findings [31], the addition of a paracrine secreting source (ADSCs in our study) increased the number of TZPs. TZPs are vital for the communication of the oocyte to its surrounding environment, exchange of low molecular weight molecules, metabolic cooperation between cumulus cells and oocytes, and regulation of meiosis [51]. Increased number of TZPs in the ADSCs co-encapsulated follicles suggests that co-encapsulation with ADSCs sustains follicular integrity and is beneficial for granulosa cell development as well as maintenance of the critical communication between the oocyte and its surrounding environment.

The findings from this study substantiate that adipose-derived stem cells can act as “feeder cells” within an in vitro culture system to provide the necessary paracrine support for folliculogenesis, thus contributing to the function and survival of granulosa and theca cells. This is also evidenced by our finding that the follicles co-encapsulated with ADSCs produced and secreted sex hormones similar to in vivo follicles [44, 45]. Ovarian follicles co-encapsulated with ADSCs secreted steroid hormones, which linearly increased with time in culture and progression of follicle growth and differentiation. Given that granulosa cells are incapable of directly producing estradiol de novo, but require androgen substrate derived from the theca cells, the successful production of A4 in our co-encapsulation system proves the presence and functional competency of theca cells, which not only serves as a substrate for E2 production but also for causing folliculotropic effects. The presence of measurable E2 in our system also provides evidence in support of successful bidirectional cross-talk between the theca and granulosa cells. Compared to steroid hormone production of murine follicles in alginate culture [16, 24], co-encapsulation with ADSCs appears to produce higher levels of A4 and markedly higher levels of P4 albeit similar levels of E2. In previously published reports of murine steroid production, P4 levels were typically less than 1 ng/ml and P4/E2 ratios of < 0.5 were representatives of normal physiologic ovarian function [34, 43, 51]. Importantly, the lower A4/E2 ratios in the larger follicles attest to the healthy status of the follicles, which is matched with improved outcomes of folliculogenesis, survival, and growth.

## Conclusion

Co-encapsulation of early-stage murine follicles with ADSCs supports the in vitro maturation of murine follicles. These in vitro matured follicles achieve meiotic competence and display similar morphologic and steroidogenic characteristics when compared to in vivo follicles. To our knowledge, this is the first report of the application of mouse ADSCs to support in vitro ovarian follicular development in a 3D co-culture setting. Research in this direction could provide the basis for future research into the secretome of ADSCs within in vitro culture system to help us better understand ADSC-derived secretory factors that play vital roles in establishing optimal ovarian micro-environment for promoting early stages of folliculogenesis. Although the scaffolding system used worked well for murine follicles, future research should also be directed at biomaterials that will allow the translation of these findings into human models. Approaches detailed in this study serves as another step toward to goal of developing fertility preservation options for pre-pubertal girls with hematologic malignancies.

## Additional files

Additional file 1: **Figure S1.** The stemness and its maintenance of ADSCs in 3D culture. A. Representative images of ADSC differentiation (scale bars = 100 μm for osteogenic and adipogenic, and scale bar = 500 μm for chondrogenic). Data presented as mean ± SEM when applicable. B. Representative images of histograms that showed positive and negative expression of mesenchymal specific stem cell markers at day 0 and after 4 days of culture. (TIF 7014 kb)

Additional file 2: **Figure S2.** Follicle survival and growth at various ADSC concentrations. There is a significant survival rate improvement for the follicles with an initial diameter of 85–95 μm at a concentration of 5.0 × 10 ^6 cells/ml. Data presented as mean ± SEM in growth curves. (TIF 3046 kb)

## Abbreviations

A4: Androstenedione
ADSCs: Adipose-derived stem cells
E2: Estradiol
GV: Germinal vesicle
MI: Meiosis 1
MII: Meiosis 2
P4: Progesterone
