# Supplementary figures and images for: Adipose-derived stem cells promote survival, growth, and maturation of early-stage murine follicles
Source: Stem Cell Res Ther. 2019 Mar 21;10:102. doi: 10.1186/s13287-019-1199-8 (PMC6427888; doi:10.1186/s13287-019-1199-8)

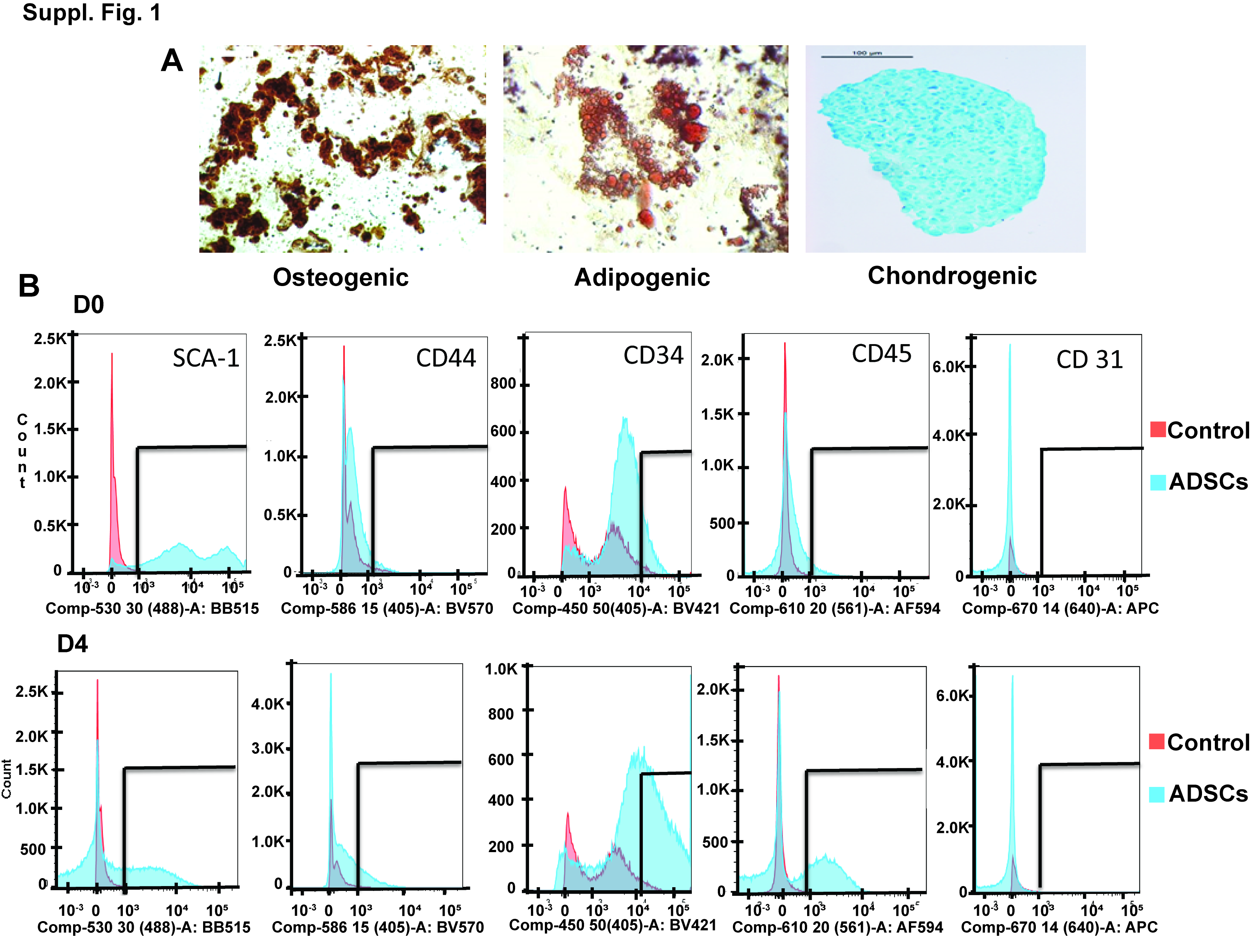

Supplement: Supplementary file 1 — Figure S1. The stemness and its maintenance of ADSCs in 3D culture. A. Representative images of ADSC differentiation (scale bars = 100 μm for osteogenic and adipogenic, and scale bar = 500 μm for chondrogenic). Data presented as mean ± SEM when applicable. B. Representative images of histograms that showed positive and negative expression of mesenchymal specific stem cell markers at day 0 and after 4 days of culture. (TIF 7014 kb) [file 13287_2019_1199_MOESM1_ESM.tif]

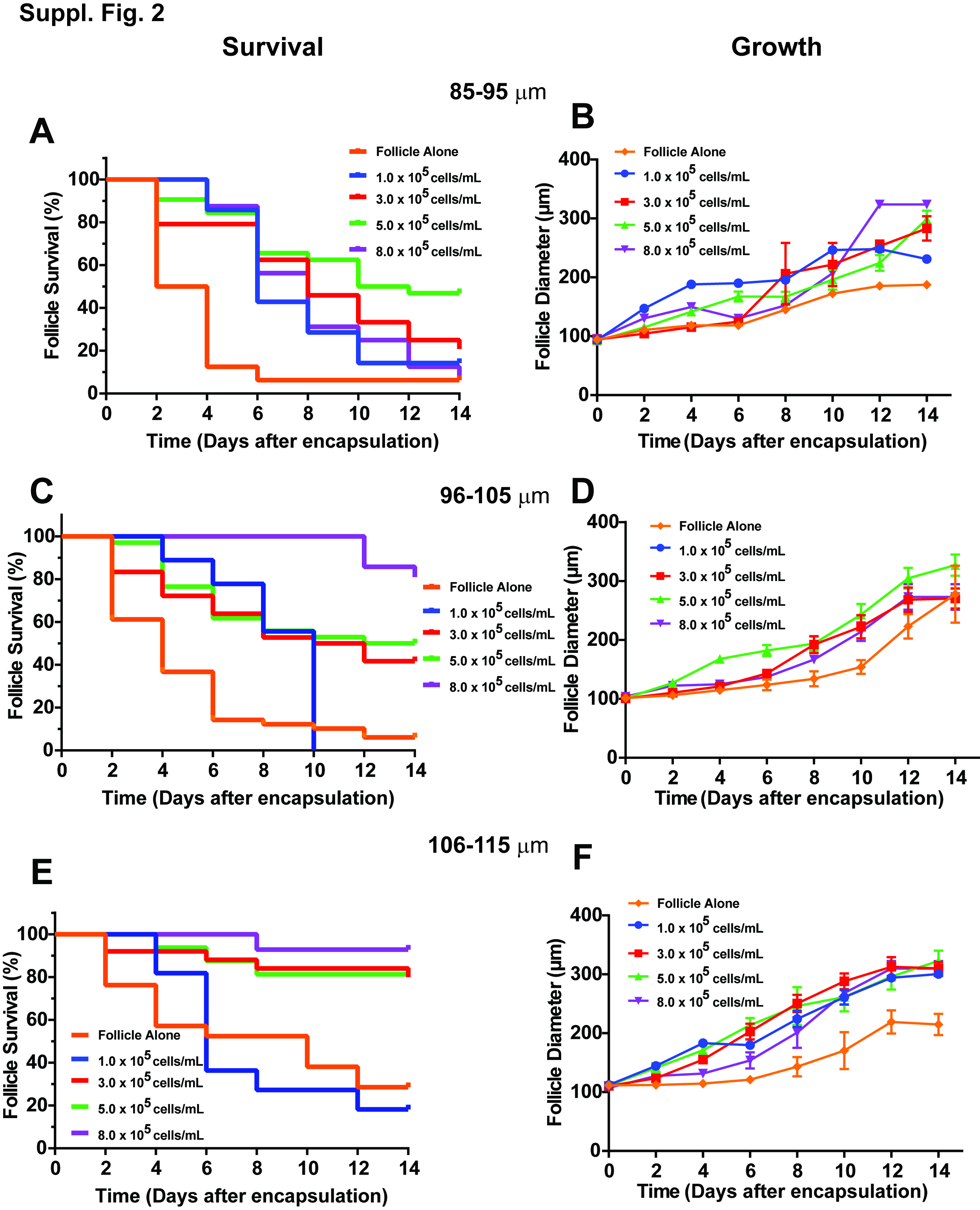

Supplement: Supplementary file 2 — Figure S2. Follicle survival and growth at various ADSC concentrations. There is a significant survival rate improvement for the follicles with an initial diameter of 85–95 μm at a concentration of 5.0 × 10 ^6 cells/ml. Data presented as mean ± SEM in growth curves. (TIF 3046 kb) [file 13287_2019_1199_MOESM2_ESM.tif]
